# Genetic variability of two ecomorphological forms of *Stenus* Latreille, 1797 in Iran, with notes on the infrageneric classification of the genus (Coleoptera, Staphylinidae, Steninae)

**DOI:** 10.3897/zookeys.626.8155

**Published:** 2016-10-20

**Authors:** Sayeh Serri, Johannes Frisch, Thomas von Rintelen

**Affiliations:** 1 Insect Taxonomy Research Department, Iranian Research Institute of Plant Protection, Agricultural Research, Education and Extension Organization, Tehran, 19395-1454, Iran; 2 Museum für Naturkunde Berlin, Leibniz-Institut für Evolutions- und Biodiversitätsforschung, Invalidenstrasse 43, D-10115 Berlin, Germany

**Keywords:** Staphylinidae, Stenus, genetic variability, ecomorphological forms, infrageneric classification, Iran

## Abstract

In this study, the genetic diversity of Iranian populations of two widespread *Stenus* species representing two ecomorphological forms, the “open living species” *Stenus erythrocnemus* Eppelsheim, 1884 and the “stratobiont” *Stenus callidus* Baudi di Selve, 1848, is presented using data from a fragment of the mitochondrial COI gene. We evaluate the mitochondrial cytochrome oxidase I haplotypes and the intraspecific genetic distance of these two species. Our results reveal a very low diversity of COI sequences in *Stenus erythrocnemus* in contrast to *Stenus callidus*. Moreover, the COI based phylogeny of a selection of Iranian *Stenus* support the monophyly of some species groups of *Stenus* proposed by Puthz (2008) and contradicts the traditional infrageneric classification.

## Introduction

Fast mutation rate and lack of recombination as well as its easy amplification and sequencing make COI a useful marker for the study of phylogeny, geographic variation and population genetics as well as species identification (Hebert et al. 2003a, b; Qian et al. 2014: 11). Many studies have demonstrated that mtDNA-COI can be used for population genetics (e.g. Szalanski et al. 2010: 8). Hajibabaei et al. (2007: 171) point out that DNA barcoding offers significant implications for the understanding of the genetic diversity of species. Here, we apply this method in the rove beetle genus *Stenus* Latreille, 1797 to test the infraspecific genetic variation of representatives of two distinct ecomorphological forms and the validity of the traditional subgeneric concept of the genus.

*Stenus* is well-known for its unique prey-capture behavior (e.g. Betz 1996: 15–34). The eversible labium, an apomorphy, and the variability of the tarsal structures seem to be responsible for the enormous radiation in this genus (Betz 2002: 1097). The labial features are involved in catching prey in a sudden manner despite the limited reaction ability of the beetle (Betz 1999: 1708). The variable tarsal morphology among the members of this genus also has adaptive values which are in accord with their habitat preferences (Betz 2006: 413–414). With about 2674 species (Puthz, unpublished), *Stenus* is one of the species-richest genera of animals in the world (Puthz 2012: 286). The members of this rove beetle clade mostly dwell in humid places such as river banks, swamps, bogs and wet grasslands. The multifunctional secretion of the pygidial glands is species-specific and acts as a survival factor against predators. This character has been used in illuminating several evolutionary trends (Schierling et al. 2013: 48, 51) and presumably is a character adaptive to the habitat where the species live (Lang et al. 2015: 22).

**Figures 1–4.**
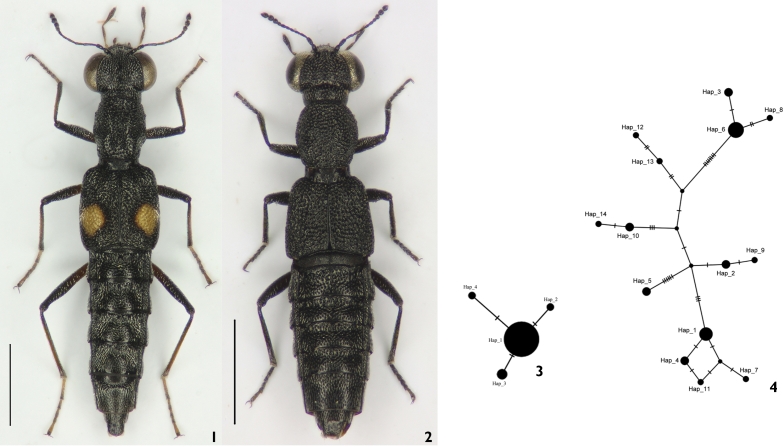
**1** *Stenus erythrocnemus* Eppelsheim, 1884. **2** *Stenus callidus* Baudi di Selve, 1848. **3** Haplotype network for cytochrome c oxidase subunit I (COI) DNA sequences of *Stenus erythrocnemus*. The circle size shows the frequency of the haplotypes. Each dashed line represents a single mutation. **4** Haplotype network for cytochrome c oxidase subunit I (COI) DNA sequences of *Stenus callidus*. The circle size shows the frequency of the haplotypes. Each dashed line represents a single mutation. Scale bars: 1mm.

In *Stenus*, two major ecomorphological forms can be distinguished, which Kastcheev and Puthz (2011: 454) termed “open-living species” with longer legs and on average bigger bodies (Figure 1), that live in habitats with less dense, often sparse vegetation such as sandy or clayey banks, and “stratobionts” with shorter legs and compact body (Figure 2), which inhabit dense vegetation structures and organic litter. Both forms are moreover distinguished by their dispersal ability, because - unlike the open-living species - there is the evolutionary tendency in stratobionts towards flightlessness. Similar morphological adaptations were already described for many rove-beetle clades such as the paederine subtribe Scopaeina Mulsant & Rey, 1878 (Frisch et al. 2002: 30). The addressed morphological characters determine the ability of the organism to colonize particular habitats and to use their resources (Betz 2006: 413). This relation between morphological features of species and ecological characteristics of habitats seems to be descriptive for niche selection. In Iran, 68 *Stenus* species were recorded (Serri and Frisch 2016: 18), among which *Stenus erythrocnemus* Eppelsheim, 1884 and *Stenus callidus* Baudi di Selve, 1848 are the most widespread across the country and were found in most provinces of Iran. According to Kastcheev and Puthz (2011: 454), *Stenus erythrocnemus* is an open-living species and *Stenus callidus* a stratobiont. Based on Iranian populations of these species, we tested the hypothesis that open-living species show a lower infraspecific genetic diversity than stratobionts owing to their higher dispersal ability.

*Stenus* was traditionally divided into subgenera according to morphological characters. Based on European species only, Rey (1884: 31) introduced the six subgenera *Hemistenus*, *Hypostenus*, *Mesostenus*, *Nestus*, *Stenus*, and *Tesnus*. Later, Heyden (1905: 262) replaced *Mesostenus* with *Parastenus* because of a homonymy with a genus in the Hymenoptera. Ádám (1987: 135), however, synonymized *Parastenus* with the older name *Hemistenus* Motschulsky, 1860, because the type species of both subgenera are considered as subjective synonyms. Therefore he introduced the new subgenus Metastenus for a distinct species group of *Hemistenus* (Herman 2001: 2041), but later he (Ádám 2001: 126) replaced this name with *Metatesnus* because of primary homonymy with *Metastenus* Walker, 1834 in the Hymenoptera. According to Puthz (2009: 47), the genus group name *Adamostenus* Özdikmen & Darılmaz, 2008, an unnecessary replacement name for *Metatesnus*, is a junior synonym of *Metatesnus*. Puthz (2001: 35) also synonymized *Nestus* with *Stenus* s. str. based on the assumption that the tarsal characters traditionally employed for these subgenera do not define distinct monophyletic groups. In the current edition of the Catalogue of Palaearctic Coleoptera, Schülke and Smetana (2015: 802–847) still divided this genus into five subgenera, which are *Hemistenus* Motschulsky, 1860, *Hypostenus* Rey, 1884, *Stenus* Latreille, 1797, *Metatesnus* Ádám, 2001 and *Tesnus* Rey, 1884. Puthz (2008: 139–148) conceived that the traditional subgeneric classification does not reflect the phylogenetic affinities within this genus and thus established 157 monophyletic species groups based on a wide range of presumed apomorphic morphological features of the species included. Ryvkin (2011: 59) argued, however, that it is better not to reject the traditional subgeneric concept prior to a comprehensive phylogenetic analysis of the subfamily. To date, there are only a few molecular studies that have investigated the phylogenetic relationships among Steninae species. The first was done recently by Koerner et al. (2013). Their results supported the monophyletic groups proposed by Puthz (2008: 139–148) and moreover revealed that some species groups of *Dianous* Leach, 1819, the second genus of the Steninae, actually constitute a monophyletic group within *Stenus*. The monophyly of some species groups proposed by Puthz (2008: 141–147) was also supported by Lang et al. (2015: 21). We performed a preliminary investigation on the intra- and interspecific genetic diversity of some Iranian *Stenus* to test the validity of the traditional classification of this genus by sequencing the “DNA Barcode” region of the mitochondrial COI gene of these species.

## Material and methods

The *Stenus* specimens this study is based on were collected in the framework of the first author’s research project on the diversity and biogeography of this genus in Iran (Serri and Frisch 2016), which was a part of a joint project between the Museum für Naturkunde Berlin and the Iranian Research Institute of Plant Protection on biodiversity and biogeography of selected insect taxa in Iran.

The specimens were collected in humid habitats such as river banks or grassland by hand collecting or sifting of gravelly soil, leaf litter and other phytodebris. Most specimens were killed with ethyl acetate, but some were directly fixated in 96% ethanol.

For DNA extraction, the abdomen of the larger species and the whole body of the smaller species were used and the DNA was purified by the CTAB method (Winnepenninckx et al. 1993). The polymerase chain reaction (PCR) was used to amplify a 5’ end fragment of the mitochondrial cytochrome c oxidase subunit I (COI) gene using the primer pair LCO1490 5’-GGTCAACAAATCATAAAGATATTGG -3’ and HCO2198 5’-TAAACTTCAGGGTGACCAAAAAATCA -3’ (Folmer et al. 1994). PCR was performed in 25µl volumes including 2.5 µM PCR buffer, 1µM MgCl2, 0.5 µM dNTP, 1 µM of each forward and reverse primers, 1µM of Taq polymerase and ddH2O up to 25 µl total volumes. In the PCR thermocycles, there was an initial denaturation step at 94° for 1.5 min, followed by 6 cycles of 94° (for 30 s) denaturation, 45° (1.5 min) annealing and 72° (for 1 min) extension and subsequently 35 cycles of 94° (for 30 s) denaturation, 51° (1.5 min) annealing and 72° (for 1 min) extension. The PCR terminated at 72° (for 5 min) for final extension. The PCR products were purified on a silica membrane with Macherey and Nagel Nucleospin kits following the manufacturer’s protocol. The purified PC products were sequenced using an ABI 3130 DNA sequencer. All sequences were aligned manually and corrected for misreads using Bioedit version 7.0.5.3 (Hall 1999). Additional mitochondrial COI GenBank sequences of *Euaesthetus ruficapillus* (Lacordaire, 1835) and *Euaesthetus superlatus* Peyerimhoff, 1937 were included in the dataset (GenBank accession numbers KM447120 and KM451370) as outgroup taxa. A Maximum Parsimony Analysis was conducted with PAUP*4.0 b10 (Swofford 2002). The dataset was also analyzed in MEGA 6 (Tamura et al. 2013) with maximum likelihood using the Tamura-Nei model with uniform rates among sites. The mean *p*-distance within each species of *Stenus callidus* and *Stenus erythrocnemus* were calculated separately using the Kimura2-parameter model (Kimura 1980) in MEGA 6. The haplotype data files of the populations of each species and the polymorphisms indices were obtained in DnaSP 5.10 (Librado and Rozas 2009) and the nexus files were transferred to PopART version 1.7 (Leigh and Bryant 2015) in order to construct a haplotype network based on the TCS algorithm (Clement et al. 2002).

## Results

The PCR amplification using LCO1490/HCO2198 primers yielded a product with a maximum length of 658 bp (excluding primers) from 91 individuals of 23 species out of a total of 157 specimens of 37 species of Iranian *Stenus*. The alignment was blasted against GenBank sequences and found to match with existing records of *Stenus*. The base composition of about 29% A, 39% T, 16% C and 16% G exhibits the common AT bias of COI.

The alignment (total of 658 bp) contained 294 variable characters, of which 246 were parsimony informative and contributed to the Maximum Parsimony (MP) Analysis. The MP Analyses produced two equally parsimonious trees with a tree length of 1197 steps, CI of 0.3768, RI of 0.8564 and RC of 0.3227 (Figure 7). Node support was estimated by bootstrap using 1000 pseudoreplicates and 100 replicates. The major clades are generally well supported (see below). All obtained sequences were submitted to GenBank (accession numbers in Table 1). The maximum likelihood tree was constructed by the heuristic search with the Nearest-Neighbor-Interchange (NNI) method, gaps treatment using all sites, the neighbor-joining (NJ) tree as the initial tree and bootstrapped with 1000 replications (Figure 8). The topology obtained from ML analyses does not deviate significantly from the MP tree. Both methods reveal a high degree of genetic homogeny among different populations of *Stenus erythrocnemus* and more pronounced heterogeny in *Stenus callidus*. The selected populations of *Stenus callidus* cluster in seven groups, but these groupings do not correspond well to the geographic distribution of the examined populations and some are not well supported in the bootstrap analysis. There are, however, some populations that form separate geographical clusters such as the populations from Kerman (specimen no. 034) with those of the Ghohrud Mountains (specimen no. 094) and the populations from Tehran Province (specimens no. 105, 107). These apparent geographical clusters are, however, not significant, because they are made up of only two populations from the same region (Figure 9). Surprisingly, the populations of *Stenus callidus* from Kordestan Province show a low similarity of the COI gene and appear in different clades of the cladogram (Figures 7, 8).

**Table 1.** The specimens used in this study with their location data and the GenBank association number of submitted sequences of COI. The specimen number codes the geographical origin of the specimens in the phylogenetic tree (Figures 7, 8).

Species	Specimen number	Collection site	GeneBank association number
***Stenus alienigenus***	147	**Kordestan**: 11 km E Sanandaj (35°20'11"N 47°09'07"E), 2100 m, 5.9.2008, leg. Serri and Frisch	KU754268
***Stenus araxis***	118	**Ardabil**: N Mt Sabalan, Gheynarjeh (38°17'18"N 47°41'22"E), 2100 m, 24.6.2008, leg. Serri	KU754251
***Stenus araxis***	121, 122	**Esfahan**: Kashan, NW Niasar, after Aznaveh (34°06'28.8"N 50°59'45.9"E), 2195 m, 19.5.2009, leg. Serri and Nasserzadeh	KU754253 KU754254
***Stenus araxis***	117	**Hamedan**: W Kabudarahang, 5 km E Goltappeh (35°12'06"N 48°14'04"E), 2210 m, 21.7.2008, leg. Serri and Nasserzadeh	KU754250
***Stenus araxis***	114	**Kordestan**: Saghez - Baneh, 27 km SW Saghez (36°08'12"N 46°02'42"E), 1600 m, 3.9.2008, leg. Serri and Frisch	KU754247
***Stenus araxis***	111	**West Azarbaijan**: W Salmas, 19 km W Kuzerash (38°11'40"N 44°33'04"E), 1960 m, 31.8.2008, leg. Serri and Frisch	KU754246
***Stenus araxis***	110	**West Azarbaijan**: Orumieh, S Silvaneh, 14 km S Ziveh (37°09'06"N 44°52'55"E), 2320 m, 1.9.2008, leg. Serri and Frisch	KU754245
**Stenus cf. araxis**	120	**Esfahan**: Natanz, S Karkas Mts, Taragh (33°24'39"N 51°46'14"E), 2580 m, 20.5.2009, leg. Serri	KU754252
**Stenus cf. araxis**	125	**Esfahan**: S Abyaneh, Bidhand (33°29'44"N 51°45'39"E), 2350 m, 18.5.2009, leg. Serri	KU754256
**Stenus cf. araxis, *Stenus araxis***	115, 116	**Tehran**: Firouzkuh, Badroud (35°48'15"N 52°39'21"E), 2060 m, 5.8.2009, leg. Serri and Nasserzadeh	KU754248 KU754249
***Stenus ater***	136	**Semnan**: NE Chashm, Hikuh, Sheil, Parvar Protected Region (36°0'54"N 53°23'07"E), 1900 m, 7.8.2009, leg. Serri and Nasserzadeh	KU754264
***Stenus brunnipes***	151	**Mazandaran**: Sari, N Mohammadabad (36°10'09"N 53°14'08"E), 820 m, 30.5.2008, leg. Serri, Nasserzadeh and Pütz	KU754270
***Stenus callidus***	089	**Chaharmahal & Bakhtiari**: Ardel, Ghahrou, Tang-e Zeverdegan (31°59'10"N 50°51'23"E), 2350 m, 23.6.2009, leg. Serri	KU754233
***Stenus callidus***	090	**Esfahan**: Chadegan, W Zayandehrud Dam (32°43'08"N 50°44'20"E), 2070 m, 20.6.2009, leg. Serri	KU754234
***Stenus callidus***	094	**Esfahan**: Kashan, S Ghamsar, Ghazaan (33°42'20"N 51°23'48"E), 2220 m, 17.5.2009, leg. Serri	KU754236
***Stenus callidus***	045, 046	**Ghazvin**: 5 km E Abgarm (35°47'53"N 49°22'43"E), 1510 m, 21.6.2004, leg. Serri and Frisch	KU754199 KU754200
***Stenus callidus***	092	**Hamedan**: Eberou road, S Emamzadeh Abdollah (34°39'20"N 48°32'19"E), 2510 m, 22.7.2008, leg. Serri and Nasserzadeh	KU754235
***Stenus callidus***	103	**Hamedan**: Shahrestaneh (34°42'56"N 48°22'21"E), 2220 m, 23.7.2008, leg. Serri and Nasserzadeh	KU754240
***Stenus callidus***	031, 033	**Hormozgan**: Siahu, Talgerdo road, Bangolan (27°50'03"N 56°28'27"E), 890 m, 19.4.2006, leg. Serri and Frisch	KU754193 KU754194
***Stenus callidus***	034	**Kerman**: Baft, 6 km N Rabor (29°20'28"N 56°50'47"E), 2640 m, 4.5.2007, leg. Serri and Frisch	KU754195
***Stenus callidus***	084	**Khuzestan**: Baghmalek, Chamkureh (31°31'42"N 49°51'55"E), 670 m, 27-28.4.2009, leg Serri	KU754231
***Stenus callidus***	079–082, 085, 086	**Kordestan**:11 km E Sanandaj (35°20'11"N 47°09'07"E), 2100 m, 5.9.2008, leg. Serri and Frisch	KU754224 KU754225 KU754226 KU754227 KU754230 KU754231
***Stenus callidus***	087	**Kordestan**: 7 km S Ghorveh, Veihaj (35°06'34"N 47°45'54"E), 2060 m, 5.9.2008, leg. Serri and Frisch	KU754232
***Stenus callidus***	098, 099	**Kordestan**: Saghez - Baneh, 27 km SW Saghez (36°08'12"N 46°02'42"E), 1600 m, 3.9.2008, leg. Serri and Frisch	KU754237 KU754238
***Stenus callidus***	035, 036	**Tehran**: Firouzkuh road, Delichai (35°40'58"N 52°28'26"E), 2000 m, 21.5.2006, leg. Serri and Frisch	KU754196 KU754197
***Stenus callidus***	105–108	**Tehran**: Firouzkuh, Badroud (35°48'15"N 52°39'21"E), 2060 m, 5.8.2009, leg. Serri and Nasserzadeh	KU754241 KU754242 KU754243 KU754244
***Stenus callidus***	100	**West Azarbaijan**: 11 km E Takht-e Soleiman (36°36'43"N 47°18'48"E), 2280 m, 7.-8.9.2008, leg. Serri and Frisch	KU754239
***Stenus callidus***	083	**West Azarbaijan**: 2 km E Takht-e Soleiman N (36°38'05"N 47°14'07"E), 2270 m, 7.-8.9.2008, leg. Serri and Frisch	KU754228
***Stenus callidus***	037	**Zanjan**: Abbar - Gilvan (36°52'50"N 48°58'32"E), 430 m, 12.7.2006, leg. Serri	KU754198
***Stenus cautus***	146	**Esfahan**: S Abyaneh, Bidhand (33°29'44"N 51°45'39"E), 2350 m, 18.5.2009, leg. Serri	KU754267
***Stenus erythrocnemus***	059, 060, 062	**Ardabil**: N Mt Sabalan, Gheynarjeh (38°17'18"N 47°41'22"E), 2100 m, 24.6.2008, leg. Serri	KU754213 KU754214 KU754215
***Stenus erythrocnemus***	024	**East Azarbaijan**: Zijenab (Mt Sahand) (37°52'08"N 46°18'46"E), 2150 m, 8.8.2005, leg. Serri and Frisch	KU754192
***Stenus erythrocnemus***	134	**Esfahan**: Natanz, Taragh, Keshe, S Mt. Karkas (33°24'39.3"N 51°46'13.9"E), 2580 m, 17.5.2009, leg. Serri	KU754262
***Stenus erythrocnemus***	070	**Gilan**: E Masuleh (37°09'48"N 49°00'19"E), 820 m, 8.6.2008, leg. Serri, Nasserzadeh and Pütz	KU754219
***Stenus erythrocnemus***	009	**Kerman**: Mahan road, 3 km S pass (30°11'29"N 57°25'42"E), 2430 m, 30.4.2007, leg. Serri and Frisch	KU754189
***Stenus erythrocnemus***	051–054	**Tehran**: Dizin (36°01'53"N 51°28'52"E), 2810 m, 10.6.2008, leg. Serri, Nasserzadeh and Pütz	KU754205 KU754206 KU754207 KU754208
***Stenus erythrocnemus***	047–050	**West Azarbaijan**: SE Makou, Gharakelisa (39°05'32"N 44°32'40"E), 1860 m, 28.8.2008, leg. Serri and Frisch	KU754201 KU754202 KU754203 KU754204
***Stenus erythrocnemus***	055–058	**West Azarbaijan**: Orumieh, S Silvaneh, 14 km S Ziveh (37°09'06"N 44°52'55"E), 2320 m, 1.9.2008, leg. Serri and Frisch	KU754209 KU754210 KU754211 KU754212
***Stenus erythrocnemus***	064	**West Azarbaijan**: 18 km W Khoy, Ghotour road (38°28'45"N 44°47'08"E), 1320 m, 29.8.2008, leg. Serri and Frisch	KU754216
***Stenus erythrocnemus***	068, 069	**West Azarbaijan**: Siahcheshmeh - Khoy, Kordkandy (N 38°55'02" E44°27'40’’), 1870 m, 28.8.2008, leg. Serri and Frisch	KU754217 KU754218
***Stenus erythrocnemus***	071–074	**West Azarbaijan**: Siahcheshmeh - Khoy, W Zarabad (N 38°44'16" E44°28'10’’), 2400 m, 30.8.2008, leg. Serri and Frisch	KU754220 KU754221 KU754222 KU754223
***Stenus erythrocnemus***	011, 012	**Yazd**: Taft, Dehbala (31°35'37"N 54°07'20"E), 2550 m, 15.5.2007, leg. Serri and Frisch	KU754190 KU754191
***Stenus fuscicornis***	156	**Mazandaran**: Ramsar, Javaherdeh road, Eshkatechal (36°50'32"N 50°34'39"E), 1450 m, 6.6.2008, leg. Serri, Nasserzadeh and Pütz	KU754272
***Stenus ganglbaueri***	153	**Mazandaran**: Baladeh, Nesen, E pass (36°14'37"N 51°27'17"E), 2960 m, 1.6.2008, leg. Serri, Nasserzadeh and Pütz	KU754271
***Stenus hypoproditor***	137	**Kordestan**: N Divandarreh, SW Zarrineh, 5 km NW Ebrahimabad (35°59'10"N 46°52'11"E), 1960 m, 4.9.2008, leg. Serri and Frisch	KU754265
***Stenus intricatus zoufali***	135	**East Azarbaijan**: Tabriz - Marand, 9 km N Amand (38°17'18"N 46°08'46"E), 1520 m, 26.8.2008, leg. Serri and Frisch	KU754263
***Stenus maculiger***	133	**West Azarbaijan**: W Salmas, 10 km W Kuzerash (38°11'40"N 44°33'04"E), 1960 m, 31.8.2008, leg. Serri and Frisch	KU754261
***Stenus martensi***	166	**Mazandaran**: Kelardasht- Marzanabad road, (36°35'39"N 51°08'37"E), 1000 m, 3.6.2008, leg. Serri, Nasserzadeh and Pütz	KU754279
***Stenus medus***	161	**Mazandaran**: Rineh, S Mt Damavand (35°53'56"N 52°06'29"E), 2960 m, 3.8.2009, leg Serri and Nasserzadeh	KU754276
***Stenus mongolicus***	138	**Semnan**: Shahroud, NE Mojem, Tash (36°31’N 54°42’E), 10.8.2009, leg. Serri and Nasserzadeh	KU754266
***Stenus ochropus***	159	**Fars**: SE Sepidan, Dalkhon (30°14'40"N 52°06'09"E), 2090 m, 9.5.2007, leg. Serri and Frisch	KU754275
***Stenus persicus***	163	**Kordestan**: Saghez - Baneh, 27 km SW Saghez (36°08'12"N 46°02'42"E), 1600 m, 3.9.2008, leg. Serri and Frisch	KU754277
***Stenus pieperi***	157	**Mazandaran**: S Salmanshahr (36°38'49"N 51°10'27"E), 280 m, 4.6.2008, leg. Serri, Nasserzadeh and Pütz	KU754273
***Stenus ressli***	158	**Mazandaran**: Tonekabon, Sehezar Forest (36°32'36"N 50°49'53"E), 1090 m, 5.6.2008, leg. Serri, Nasserzadeh and Pütz	KU754274
***Stenus schah***	164	**Kohgiluye & Boyerahmad**: N Yasuj, Sepidar, Dilgan River (30°45'03"N 51°08'07"E), 2270 m, 18.6.2009, leg. Serri	KU754278
***Stenus turk***	124	**Esfahan**: S Abyaneh, Bidhand (33°29'44"N 51°45'39"E), 2350 m, 18.5.2009, leg. Serri	KU754255
***Stenus turk***	126–129	**Golestan**: NE Kalaleh, Zav, Totlitamak village (37°29'36"N 55°46'25"E), 1240 m, , 16.10.2009, leg. Serri	KU754257 KU754258 KU754259 KU754260
***Stenus viti***	148	**Mazandaran**: Kelardasht - Marzanabad (36°35'40"N 51°08'37"E), 1000 m, 3.6.2008, leg. Serri, Nasserzadeh and Pütz	KU754269

The haplotype networks for COI of *Stenus callidus* and *Stenus erythrocnemus* (Figures 3, 4) comprise fourteen and four haplotypes, respectively. Haplotype diversity (h) was estimated at 0.911±0.034 for *Stenus callidus* and 0.267±0.107 for *Stenus erythrocnemus*. The nucleotide diversity (π i) of each species was calculated as 0.01348±0.00074 for *Stenus callidus* and 0.00045±0.00019 for *Stenus erythrocnemus* (Table 4). In *Stenus callidus*, no haplotype has an outstandingly high frequency, while *Stenus erythrocnemus* has a dominant haplotype (Hap_1) found in populations of the Elburz and Zagros Mountains and the central mountain ranges.

The maximum genetic distance among populations does not exceed 0.003% in *Stenus erythrocnemus* and is much higher in *Stenus callidus* with 0.028% (Tables 2, 3). The highest genetic distance as well as the highest haplotype diversity in the populations of *Stenus callidus* was observed in the central zone of the Zagros Mountains. In *Stenus erythrocnemus*, the highest genetic distance is among the populations of northwestern Iran.

Regarding the subgeneric concept of *Stenus*, our results (Figures 7, 8) do not support the traditional grouping except for *Hemistenus*, the selected species of which appear in the same clade. Our results rather support the monophyly of those species groups of Puthz (2008: 139–148), which we tested with at least two representatives. These species groups and the included species are: *Stenus guttula* group with *Stenus erythrocnemus* and *Stenus maculiger*, *Stenus cordatus* group with *Stenus araxis* and *Stenus turk*, *Stenus glacialis* group with *Stenus medus*, *Stenus persicus* and *Stenus schah*, *Stenus ochropus-ludyi-coarcticollis* group with *Stenus martensi*, *Stenus ochropus*, *Stenus pieperi* and *Stenus ressli*, *Stenus ater* group with *Stenus ater*, *Stenus hypoproditor* and *Stenus intricatus zoufali*.

**Table 2.** Kimura two-parameter pairwise genetic distances between populations of *Stenus callidus*.

	031	033	034	035	036	037	045	046	079	080	081	082	083	084	085	086	087	089	090	092	094	098	099	100	103	105	106	107	108
031																													
033	0.000																												
034	0.006	0.006																											
035	0.022	0.022	0.022																										
036	0.022	0.022	0.022	0.000																									
037	0.000	0.000	0.006	0.022	0.022																								
045	0.002	0.002	0.008	0.023	0.023	0.002																							
046	0.002	0.002	0.008	0.023	0.023	0.002	0.000																						
079	0.012	0.012	0.011	0.028	0.028	0.012	0.014	0.014																					
080	0.020	0.020	0.020	0.002	0.002	0.020	0.022	0.022	0.026																				
081	0.006	0.006	0.000	0.022	0.022	0.006	0.008	0.008	0.011	0.020																			
082	0.012	0.012	0.011	0.028	0.028	0.012	0.014	0.014	0.000	0.026	0.011																		
083	0.020	0.020	0.020	0.002	0.002	0.020	0.022	0.022	0.026	0.000	0.020	0.026																	
084	0.020	0.020	0.020	0.002	0.002	0.020	0.022	0.022	0.026	0.000	0.020	0.026	0.000																
085	0.000	0.000	0.006	0.022	0.022	0.000	0.002	0.002	0.012	0.020	0.006	0.012	0.020	0.020															
086	0.000	0.000	0.006	0.022	0.022	0.000	0.002	0.002	0.012	0.020	0.006	0.012	0.020	0.020	0.000														
087	0.003	0.003	0.009	0.022	0.022	0.003	0.005	0.005	0.015	0.020	0.009	0.015	0.020	0.020	0.003	0.003													
089	0.020	0.020	0.020	0.002	0.002	0.020	0.022	0.022	0.026	0.000	0.020	0.026	0.000	0.000	0.020	0.020	0.020												
090	0.020	0.020	0.020	0.002	0.002	0.020	0.022	0.022	0.026	0.000	0.020	0.026	0.000	0.000	0.020	0.020	0.020	0.000											
092	0.023	0.023	0.023	0.005	0.005	0.023	0.025	0.025	0.030	0.003	0.023	0.030	0.003	0.003	0.023	0.023	0.023	0.003	0.003										
094	0.008	0.008	0.002	0.023	0.023	0.008	0.009	0.009	0.012	0.022	0.002	0.012	0.022	0.022	0.008	0.008	0.011	0.022	0.022	0.022									
098	0.011	0.011	0.008	0.020	0.020	0.011	0.012	0.012	0.014	0.019	0.008	0.014	0.019	0.019	0.011	0.011	0.014	0.019	0.019	0.022	0.009								
099	0.003	0.003	0.009	0.022	0.022	0.003	0.002	0.002	0.015	0.020	0.009	0.015	0.020	0.020	0.003	0.003	0.003	0.020	0.020	0.023	0.011	0.014							
100	0.020	0.020	0.020	0.002	0.002	0.020	0.022	0.022	0.026	0.000	0.020	0.026	0.000	0.000	0.020	0.020	0.020	0.000	0.000	0.003	0.022	0.019	0.020						
103	0.020	0.020	0.020	0.002	0.002	0.020	0.022	0.022	0.026	0.000	0.020	0.026	0.000	0.000	0.020	0.020	0.020	0.000	0.000	0.003	0.022	0.019	0.020	0.000					
105	0.014	0.014	0.011	0.020	0.020	0.014	0.015	0.015	0.017	0.019	0.011	0.017	0.019	0.019	0.014	0.014	0.014	0.019	0.019	0.022	0.012	0.012	0.014	0.019	0.019				
106	0.011	0.011	0.008	0.020	0.020	0.011	0.012	0.012	0.014	0.019	0.008	0.014	0.019	0.019	0.011	0.011	0.014	0.019	0.019	0.022	0.009	0.000	0.014	0.019	0.019	0.012			
107	0.011	0.011	0.008	0.017	0.017	0.011	0.012	0.012	0.014	0.015	0.008	0.014	0.015	0.015	0.011	0.011	0.011	0.015	0.015	0.019	0.009	0.009	0.011	0.015	0.015	0.003	0.009		
108	0.012	0.012	0.009	0.022	0.022	0.012	0.014	0.014	0.015	0.020	0.009	0.015	0.020	0.020	0.012	0.012	0.015	0.020	0.020	0.023	0.011	0.002	0.015	0.020	0.020	0.014	0.002	0.011	

**Table 3.** Kimura two-parameter pairwise genetic distances between populations of *Stenus erythrocnemus*.

	009	011	012	024	047	048	049	050	051	052	053	054	055	056	057	058	059	060	062	064	068	069	070	071	072	073	074	134
009																												
011	0.000																											
012	0.000	0.000																										
024	0.002	0.002	0.002																									
047	0.000	0.000	0.000	0.002																								
048	0.000	0.000	0.000	0.002	0.000																							
049	0.002	0.002	0.002	0.003	0.002	0.002																						
050	0.000	0.000	0.000	0.002	0.000	0.000	0.002																					
051	0.000	0.000	0.000	0.002	0.000	0.000	0.002	0.000																				
052	0.000	0.000	0.000	0.002	0.000	0.000	0.002	0.000	0.000																			
053	0.000	0.000	0.000	0.002	0.000	0.000	0.002	0.000	0.000	0.000																		
054	0.000	0.000	0.000	0.002	0.000	0.000	0.002	0.000	0.000	0.000	0.000																	
055	0.000	0.000	0.000	0.002	0.000	0.000	0.002	0.000	0.000	0.000	0.000	0.000																
056	0.000	0.000	0.000	0.002	0.000	0.000	0.002	0.000	0.000	0.000	0.000	0.000	0.000															
057	0.000	0.000	0.000	0.002	0.000	0.000	0.002	0.000	0.000	0.000	0.000	0.000	0.000	0.000														
058	0.000	0.000	0.000	0.002	0.000	0.000	0.002	0.000	0.000	0.000	0.000	0.000	0.000	0.000	0.000													
059	0.000	0.000	0.000	0.002	0.000	0.000	0.002	0.000	0.000	0.000	0.000	0.000	0.000	0.000	0.000	0.000												
060	0.002	0.002	0.002	0.003	0.002	0.002	0.003	0.002	0.002	0.002	0.002	0.002	0.002	0.002	0.002	0.002	0.002											
062	0.002	0.002	0.002	0.003	0.002	0.002	0.000	0.002	0.002	0.002	0.002	0.002	0.002	0.002	0.002	0.002	0.002	0.003										
064	0.000	0.000	0.000	0.002	0.000	0.000	0.002	0.000	0.000	0.000	0.000	0.000	0.000	0.000	0.000	0.000	0.000	0.002	0.002									
068	0.000	0.000	0.000	0.002	0.000	0.000	0.002	0.000	0.000	0.000	0.000	0.000	0.000	0.000	0.000	0.000	0.000	0.002	0.002	0.000								
069	0.000	0.000	0.000	0.002	0.000	0.000	0.002	0.000	0.000	0.000	0.000	0.000	0.000	0.000	0.000	0.000	0.000	0.002	0.002	0.000	0.000							
070	0.000	0.000	0.000	0.002	0.000	0.000	0.002	0.000	0.000	0.000	0.000	0.000	0.000	0.000	0.000	0.000	0.000	0.002	0.002	0.000	0.000	0.000						
071	0.000	0.000	0.000	0.002	0.000	0.000	0.002	0.000	0.000	0.000	0.000	0.000	0.000	0.000	0.000	0.000	0.000	0.002	0.002	0.000	0.000	0.000	0.000					
072	0.000	0.000	0.000	0.002	0.000	0.000	0.002	0.000	0.000	0.000	0.000	0.000	0.000	0.000	0.000	0.000	0.000	0.002	0.002	0.000	0.000	0.000	0.000	0.000				
073	0.000	0.000	0.000	0.002	0.000	0.000	0.002	0.000	0.000	0.000	0.000	0.000	0.000	0.000	0.000	0.000	0.000	0.002	0.002	0.000	0.000	0.000	0.000	0.000	0.000			
074	0.000	0.000	0.000	0.002	0.000	0.000	0.002	0.000	0.000	0.000	0.000	0.000	0.000	0.000	0.000	0.000	0.000	0.002	0.002	0.000	0.000	0.000	0.000	0.000	0.000	0.000		
134	0.000	0.000	0.000	0.002	0.000	0.000	0.002	0.000	0.000	0.000	0.000	0.000	0.000	0.000	0.000	0.000	0.000	0.002	0.002	0.000	0.000	0.000	0.000	0.000	0.000	0.000	0.000	

**Table 4.** Summary of genetic diversity indices in the mitochondrial COI gene segment of *Stenus callidus* and *Stenus erythrocnemus*.

Species	N	L	k	H	h (±standard deviation)	π (±standard deviation)	Haplotype no.: sequence(s) no.
*Stenus callidus*	29	658	30	14	0.911±0.034	0.01348±0.00074	Hap_1: 031, 033, 037, 085, 086 Hap_2: 034, 081 Hap_3: 035, 036 Hap_4: 045, 046 Hap_5: 079, 082 Hap_6: 080, 083, 084, 089, 090,100, 103 Hap_7: 087 Hap_8: 092 Hap_9: 094 Hap_10: 098,106 Hap_11: 099 Hap_12: 105 Hap_13: 107 Hap_14: 108
*Stenus erythrocnemus*	28	658	3	4	0.267±0.107	0.00045±0.00019	Hap_1: 009, 011, 012, 047, 048, 050, 051, 052, 053, 054, 055, 056, 057, 058, 059, 064, 068, 069, 070, 071, 072, 073, 074, 134 Hap_2: 024 Hap_3: 049, 062 Hap_4: 060

Abbreviations: N , number of sequences ; L , sequence length (number of bases) ; k , number of variable sites ; H , number of haplotypes ; h , haplotype diversity ; π , nucleotide diversity .

## Discussion

With the example of Iranian populations of the open-living *Stenus erythrocnemus* and the stratobiont *Stenus callidus*, we demonstrate that different ecomorphological forms of congeneric species with differing dispersal ability and degree of geneflow can show a different degree of infraspecific genetic variability.

**Figure 5.**
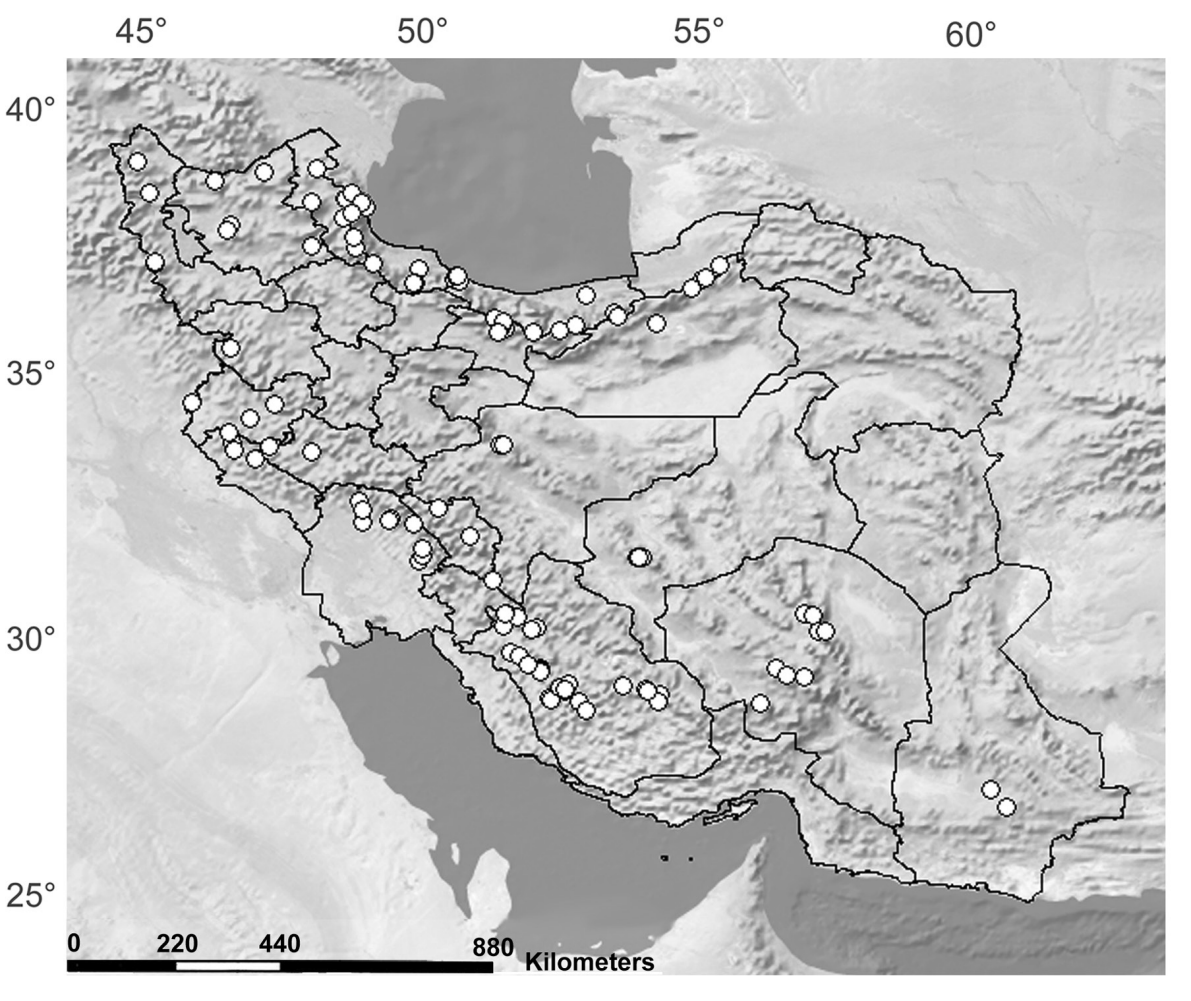
Distribution of *Stenus erythrocnemus* in Iran (after Serri and Frisch 2016: 28).

The open-living *Stenus erythrocnemus* is the most widespread *Stenus* in Iran. It was found in most of the country in high abundance at elevations between 250 m and 2800 m a.s. l. (Figure 5, after Serri and Frisch 2016: 27). As an example of the open-living ecomorphological form described by Kastcheev and Puthz (2011: 454), this mobile species does not show geographically structured populations. The low level of haplotype diversity as well as the low intraspecific distance of this species indicate a high level of gene flow between the populations of this species, which are connected to each other even across zoogeographic barriers due to the species’ dispersal ability. This gene flow within the Iranian meta-population of *Stenus erythrocnemus* is probably supported by the wide ecological adaptability, which prevents geographic isolation. Unlike the remainder of Iranian *Stenus*, we repeatedly collected *Stenus erythrocnemus* not only in natural habitats, but also in polluted sites and anthropogenic places such as watering channels of farms far from natural, permanent watercourses.

**Figure 6.**
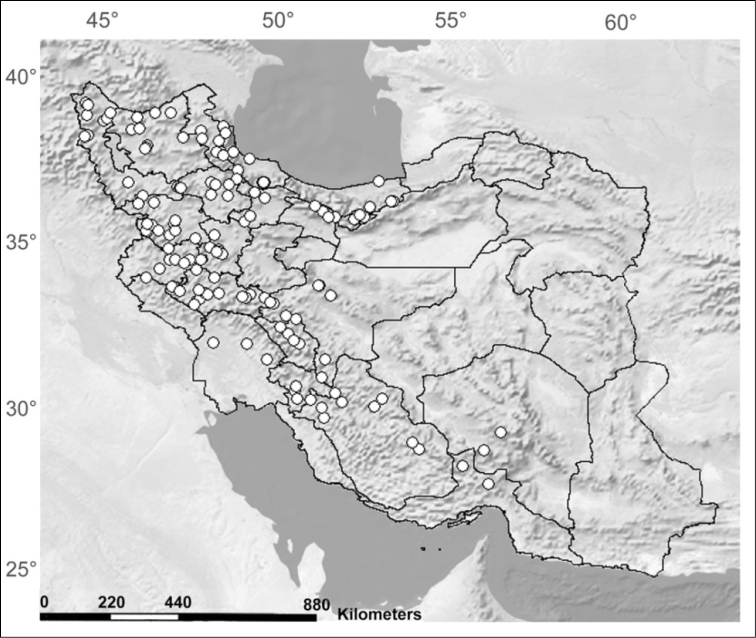
Distribution of *Stenus callidus* in Iran (after Serri and Frisch 2016: 28).

The stratobiont *Stenus callidus*, the second widespread *Stenus* in Iran, was collected in high abundance in most of the collecting sites all over the country (Figure 6, after Serri and Frisch 2016: 27). Our cladogram shows the separation of the tested *Stenus callidus* populations into six genetic units, which can be explained by the limited dispersal ability of the mostly micropterous individuals of *Stenus callidus*. The genetic variability of *Stenus callidus*, as shown by the higher genetic distance among populations and more diverse haplotypes, might moreover be increased by discontinuity of suitable habitats caused by man-made destruction, because – in contrast to *Stenus erythrocnemus* – the species usually avoids strongly disturbed sites.

**Figure 7.**
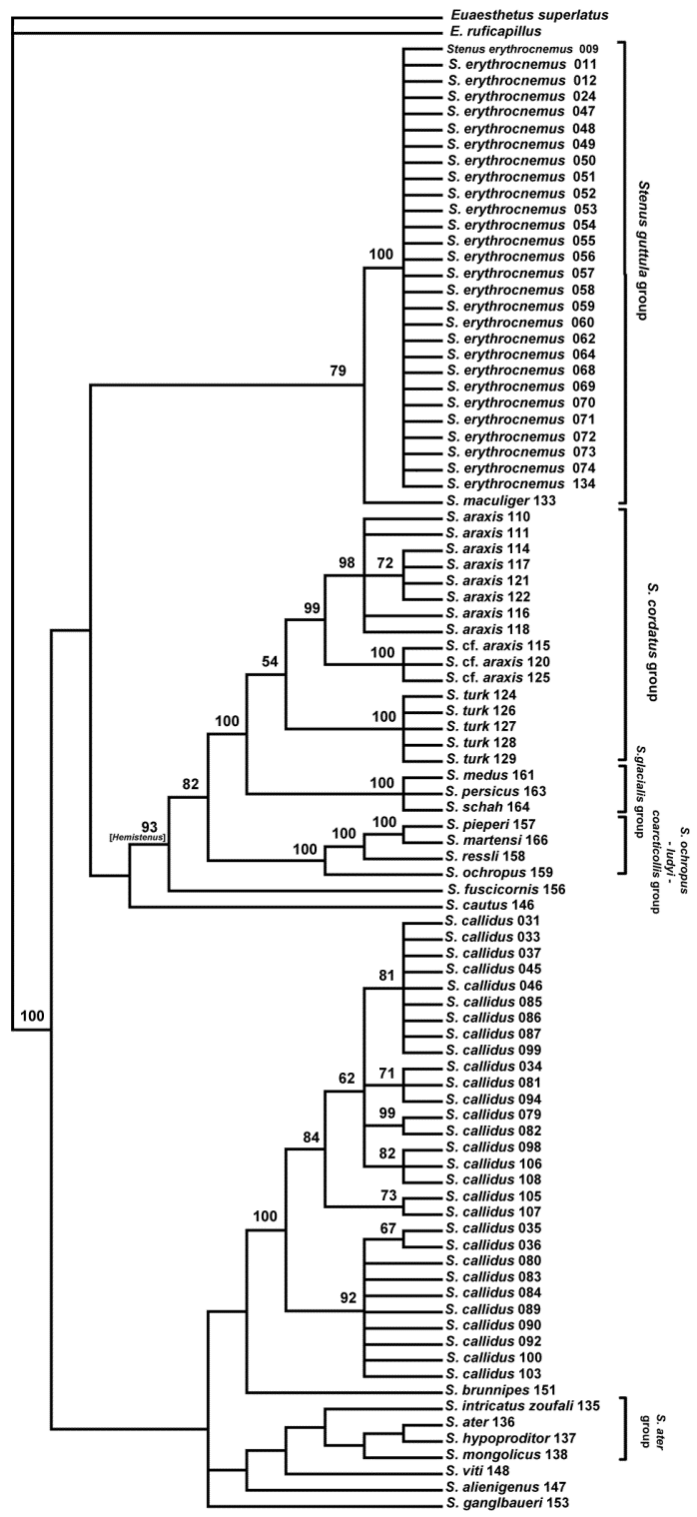
Strict consensus of most-parsimonious trees. Values above the branches indicate clade bootstrap support (>50) using 1000 replicates. The geographical origin of the specimens is coded by numbers behind the species name which correspond to the geographical information in Table 1.

**Figure 8.**
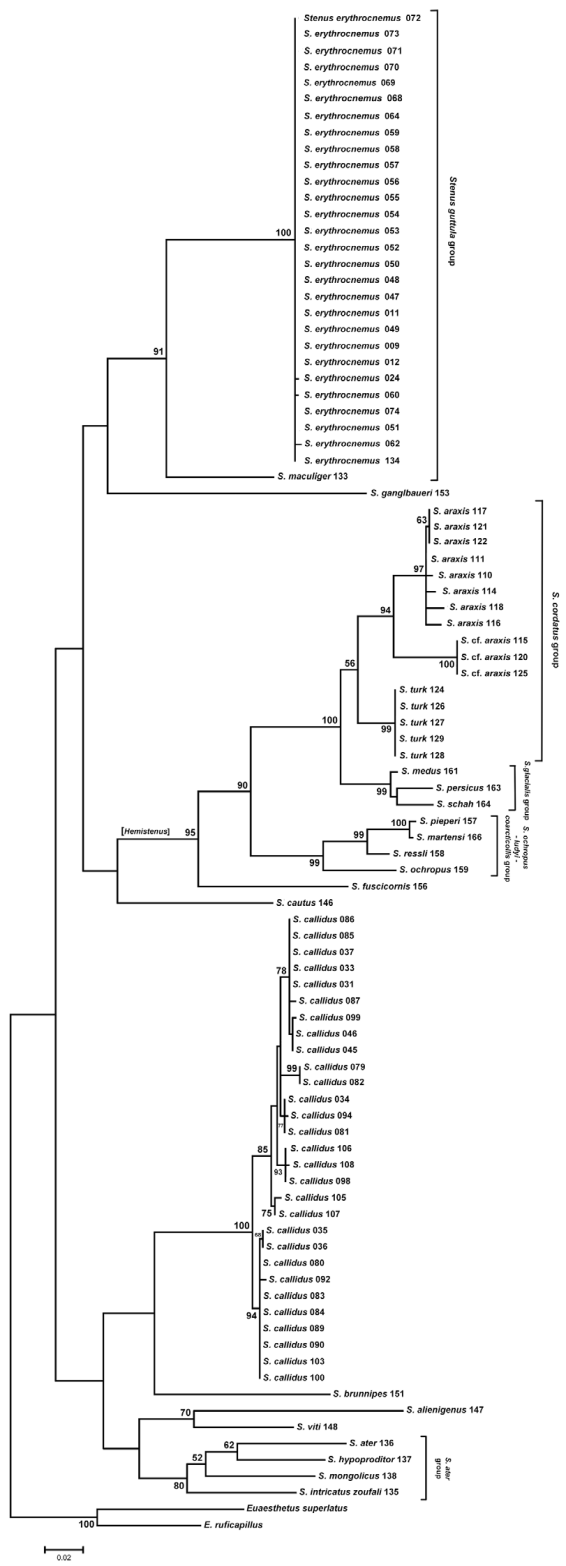
Maximum likelihood phylogram. Numbers on branches are bootstrap values (>50). The specimen codes correspond to the geographical information in Table 1. Scale shows number of substitutions per site.

**Figure 9.**
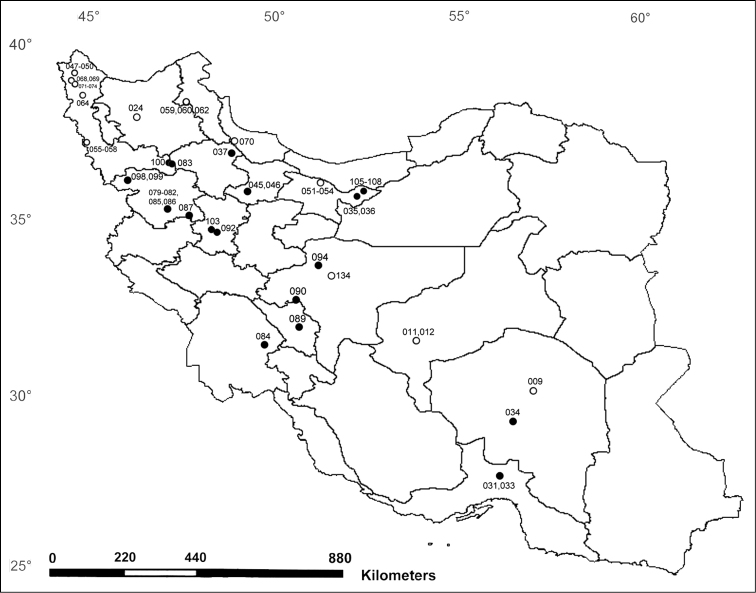
Distribution map of sequenced specimens of *Stenus callidus* (●) and *Stenus erythrocnemus* (○). Numbers are haplotype numbers (see Table 4). Sites with more than one haplotype number indicate several geographically close localities.

Though our COI examination of a limited number of West Palaearctic species of *Stenus* is not extensive when it comes to understanding the supraspecific phylogeny of the entire clade, it clearly shows the monophyly of the included *Hemistenus* species and the polyphyletic relationship among the investigated members of subgenus Stenus. The relationships of *Tesnus* and *Metatesnus* with other species were not resolved, because we were able to extract DNA from only one species of each of these subgenera. The monophyly of the selected *Hemistenus* species is, however, consistent with the result of the analysis performed by Koerner et al. (2013: 340).

Our results, which agree with those of Koerner et al. (2013: 345) and Lang et al. (2015: 20–21), further support the monophyly of the tested infrageneric species groups proposed by Puthz (2008: 139–148). On one hand, this result is not very significant, as only few species of some of these groupings were included in this study. On the other hand, our results clearly contradict the traditional subgeneric concept, which is followed until today, and proves the morphological characters this erroneous concept is based on to be phylogenetically uninformative convergencies. The included members of one of these traditional subgenera, *Hemistenus*, constitute, however, one well supported clade (bootstrap value >90) comprising the *Stenus cordatus* group, the *Stenus glacialis* group and the *Stenus ochropus-ludyi-coarcticollis* group. Particularly the first two species groups are closely related sister groups (bootstrap value 100). Further investigations are necessary to show whether *Hemistenus* – unlike the other traditional subgenera - actually represents a monophyletic group or not.

Our results support the supraspecific phylogenetic concept of Puthz (2008: 139–148) and at the same time largely contradict the traditional subgenera. Therefore, these subgenera should not be used anymore in favour of the informal species groups, though the monophyly of some of them still has to be proved.

Among the collected specimens of *Stenus araxis*, there are specimens which show differences in the structure of the median lobe of the aedeagus and in the spermatheca. The cladogram shows that these specimens form a separate clade although they have no geographic separation. Both morphological and genetic examination of a broader basis of specimens is necessary to clarify whether this form should be considered as a distinct species.

Since we did not succeed in extracting DNA from a large number of the recently collected species or from the Iranian material in Scheerpeltz solution collected by Senglet, it was not possible to include all Iranian species into the analysis. Moreover, the paucity of fresh specimens of many rare species did not allow us to use genetic data of these species in our phylogenetic analysis. Nevertheless, this preliminary study provides benchmark data for future phylogenetic investigations that include a higher number of taxa at a wider geographic scale and additional genes. Our current analysis based on a COI fragment suggests that the ‘barcoding fragment’ studied here can also be used for testing the phylogenetic validity of supraspecific groups.
